# Transcription factor *ASCL1* targets *SLC6A13* to inhibit the progression of hepatocellular carcinoma via the glycine-inflammasome signaling

**DOI:** 10.17305/bb.2024.10328

**Published:** 2024-12-01

**Authors:** Hongyan Zhang, Ruiqing Zong, Huiqi Wu, Jun Jiang, Chuanyong Liu, Suiyi Liu

**Affiliations:** 1 Department of Intensive Care Medicine, The Third Hospital Affiliated to Naval Medical University, Jiading District, Shanghai, China; 2 State Key Laboratory of Genetic Engineering, Shanghai Engineering Research Center of Industrial Microorganisms, School of Life Sciences, Fudan University, Shanghai, China; 3 Department of Medical Services, The First Hospital Affiliated to Naval Medical University, Yangpu District, Shanghai, China; 4 Department of Medical Engineering, The Third Hospital Affiliated to Naval Medical University, Jiading District, Shanghai, China

**Keywords:** Solute carrier family 6 member 13 (*SLC6A13*), hepatocellular carcinoma (HCC), transcription factor, achaete-scute family bHLH transcription factor 1 (*ASCL1*), glycine-inflammasome signaling.

## Abstract

Hepatocellular carcinoma (HCC), the most common primary liver cancer, typically arises from chronic liver conditions such as hepatitis, cirrhosis, or other chronic liver diseases, and is characterized by its aggressive nature and poor prognosis. The purpose of this research was to clarify the function of achaete-scute family bHLH transcription factor 1 (ASCL1) and solute carrier family 6 member 13 (SLC6A13) in influencing tumor cell behavior, inflammatory responses, and the regulation of inflammasomes. We analyzed the differentially expressed genes (DEGs) in the Cancer Genome Atlas-Liver Hepatocellular Carcinoma (TCGA-LIHC) database, as well as in the GSE14520 and GSE67764 datasets, to identify the expression changes of SLC6A13 in liver cancer. The prognostic significance of SLC6A13 in LIHC was assessed through Kaplan-Meier survival curve analysis. Transcriptional regulation of SLC6A13 by ASCL1 was explored using the Joint Annotation of the Human Genome and other species by the Systematic Pipeline for the Annotation of Regulatory Regions (JASPAR) database and dual-luciferase assays. In vitro experiments investigated the impact of ASCL1 and SLC6A13 overexpression on HCC cell growth. Additionally, the effects of ethanol treatment and glycine modulation on the inflammatory response in HCC cell lines were evaluated. HCC samples showed reduced levels of SLC6A13, which correlates with a better prognosis for liver metastases. Elevated SLC6A13 expression correlated with improved overall survival (OS), progression-free survival (PFS), recurrence-free survival (RFS), and disease-specific survival (DSS). ASCL1 upregulated SLC6A13 and inhibited proliferation, migration, and invasion of HCC cells. Ethanol induced the production of inflammatory cytokines, which was enhanced by overexpression of SLC6A13 but counteracted by glycine. This study highlighted elevated expression of SLC6A13 in LIHC which has been correlated with improved OS, PFS, RFS, and DSS. Overexpression of SLC6A13 and ASCL1 in HCC cells enhanced inflammasome activation, which was exacerbated by ethanol and attenuated by glycine.

## Introduction

Around 95% of primary liver malignancies are attributed to hepatocellular carcinoma (HCC), the most prevalent primary liver malignancy [1, 2]. The onset of liver cancer is insidious and has no obvious specificity in the early stage [3]. As the disease progresses, patients may experience pain, abdominal swelling, fatigue, and a mass in the upper abdomen [4, 5]. Other symptoms that can occur in patients with liver cancer include low-grade fever, jaundice, diarrhea, and upper gastrointestinal bleeding [6, 7]. According to research, liver cancer can induce a variety of different disorders, such as hepatic encephalopathy, liver failure, metastatic liver cancer, and so on [8, 9]. Currently, risk factors include hepatitis B and C viruses, among others [10, 11]. Since the etiology of HCC is intricate and varied, there is now no effective treatment, even though several research studies are carried out annually on the care of HCC patients. Targeted medicines have been approved as a result of recent developments in the molecular pathophysiology of HCC, which have also helped identify possible therapeutic targets. Therefore, the search for reliable biomarkers for early detection, prognosis, and treatment response is a key area of ongoing research.

Inflammation, characterized by an increase in low-grade chronic inflammation with age, serves as a hallmark of aging and is closely associated with various age-related diseases, including chronic liver disease (CLD) and HCC [12]. It was a complex biological response to harmful stimuli and is essential to many physiological and pathological processes [13]. In the context of HCC, inflammation has garnered significant attention due to its multifaceted impact on tumorigenesis and progression. It is associated with various chronic inflammatory and fibrotic diseases, including hepatitis B, hepatitis C, alcoholic liver disease, and nonalcoholic fatty liver disease [14]. Of these, tumor necrosis factor-alpha (TNF-α) is intimately associated with the development and course of HCC. It can also stimulate the development of HCC by modulating the immune system and acting as a primary mediator of inflammation [15]. When inflammasomes like NLR family pyrin domain containing 3 (NLRP3) are activated, proinflammatory cytokines like interleukin-1 beta (IL-1β) and interleukin-18 (IL-18) are secreted, which can cause a persistent inflammatory state that worsens liver damage and accelerates the growth of cancer [16]. In the meanwhile, the control of HCC development has been linked to several important inflammatory mediators, such as nuclear factor-κB (NF-κB) and NLRP3 inflammasome. The study conducted by Zuo et al. [17] sheds insight on the function of NF-kappaB, a major regulator of genes linked to inflammation and tumorigenesis, in controlling the expression of telomerase reverse transcriptase (hTERT), an essential component of the pathophysiology of colorectal cancer. Additionally, the study by Zhu et al. emphasizes the usefulness of the inflammation-nutrition scope (INS), which integrates indicators of nutritional status and systemic inflammation, as an independent predictive predictor for HCC, particularly in its early phases [18]. This offers crucial guidance for clinical trials focusing on nutritional interventions in HCC patients. Furthermore, the study by Zhu et al. affirmed the key role of inflammation in the occurrence and progression of HCC, emphasizing that annexin A3 (*ANXA3*) is involved in reshaping the immune microenvironment by upregulating the infiltrating neutrophil–lymphocyte ratio (iNLR), thereby promoting tumorigenesis in HCC [19]. Research has found that RCC1 domain-containing protein 2 (HERC2) enhances the stemness of HCC cells and PD-L1-mediated immune escape, which is associated with the activation of the signal transducer and activator of transcription 3 (STAT3) pathway during the inflammation–cancer transition. Overexpression of HERC2 promotes tumor development and progression in an orthotopic HCC model [20]. In light of these observations, the current state of research on inflammation in HCC underscores its paramount significance and potential as a therapeutic target.

Solute carrier family 6 (SLC6) is a group of transport proteins involved in substrate translocation across cell membranes, and several studies have proposed a potential link between this family of proteins and HCC [21, 22]. For example, certain SLC6 transporters are responsible for the uptake of amino acids, and dysregulation of amino acid transport can promote the occurrence of tumors such as HCC by changing cell metabolism and the tumor microenvironment [23]. The miR-212-3p/solute carrier family 6 member 1 (*SLC6A1*) axis has been found by Zhang et al. [24] as a possible predictive model in HCC, highlighting its significance as a therapeutic target for this illness. *SLC6A1* may play a role in the development and prognosis of HCC, according to different research by Zhang et al. [25]. This reveals that this protein is closely associated with HCC survival and prognosis within the Myc-related competitive endogenous RNA (ceRNA) network. Solute carrier family 6 member 13 (*SLC6A13*) is a member of SLC6 [26]. Tran et al. [27] mentioned that solute carrier family 6 member 6 (*SLC6A6*) and *SLC6A13* are involved in the uptake of δ-aminolevulinic acid (ALA) by cancer cells and enhance the accumulation of protoporphyrin induced by ALA. This is critical for the efficacy of photodynamic therapy in treating cancers, such as colon and epithelial cancers. Through research on carotid paragangliomas (CPGLs), Snezhkina et al. [28] found that *SLC6A13* can be used as one of the candidate genes and may be involved in the occurrence and progression of neuroendocrine (NE) tumors. However, at present, the connection between *SLC6A13* and HCC is unclear and requires further research.

The dysregulation of *SLC6A13* and its association with HCC inflammation provide new avenues for exploring new therapeutic avenues. Given the complex interactions between HCC, inflammation, and cancer progression, our study aims to uncover the complex mechanisms underlying HCC pathogenesis and investigate the role of *SLC6A13* and achaete-scute family bHLH transcription factor 1 (*ASCL1)* genes in inflammasome activation, amino acid metabolism, and inflammatory response regulation in HCC. By elucidating the interregulatory role of these genes, particularly in the context of ethanol and glycine therapy, this study will advance our understanding of HCC pathogenesis.

## Materials and methods

### Dataset download and screening of differentially expressed genes (DEGs)

In this study, we accessed and processed gene expression data from two different sources: the Gene Expression Omnibus (GEO, https://www.ncbi.nlm.nih.gov/gds/) database, and The Cancer Genome Atlas (TCGA) database provided by the Sangerbox website (http://vip.sangerbox.com/home.html). The GEO database provided the GSE14520 dataset, which comprised 220 matched normal controls and 225 liver tumor samples. Similarly, the GSE67764 dataset, obtained from the GEO database, includes three human HCC tissues and six human normal liver tissues. Fifty normal samples and 371 LIHC samples were accessed in the TCGA database. The “limma” package from the R programming language was used to identify DEGs. A fold change (FC) threshold of >1.5 for upregulated genes and 0.67 for downregulated genes was used to evaluate statistical significance, using a minimum *P* value of less than 0.05 to assess statistical significance.

### Bioinformatics analysis of *SLC6A13* expression and prognosis in TCGA-LIHC

The expression profile of *SLC6A13* was assessed through rigorous bioinformatic analyses using diverse datasets and computational tools. First, the Wilcox. test was used to detect the expression of *SLC6A13* in TCGA-LIHC. Additionally, the expression of *SLC6A13* in tumor and normal samples in the GSE145290 and GSE67764 datasets was also examined using the R package. Subsequently, the prognostic significance of differential expression of *SLC6A13* in TCGA-LIHC patients was analyzed by the Kaplan–Meier (KM) plotter website (https://kmplot.com/). KM survival curves were generated for overall survival (OS), progression-free survival (PFS), recurrence-free survival (RFS), and disease-specific survival (DSS). The log-rank test was used to obtain *P* values in order to assess the statistical significance of survival differences. To further delineate the clinical relevance of *SLC6A13* expression, the comprehensive cancer data mining platform UALCAN (https://ualcan.path.uab.edu/) was utilized. The expression patterns of *SLC6A13* across various clinical features within the TCGA-LIHC cohort were analyzed, including the patient’s gender, nodal metastasis status, tumor grade, individual cancer stages, Histological subtypes, and TP53 mutation status.

### Comprehensive analysis of JASPAR database and *ASCL1* expression patterns

To predict the potential binding motifs of *ASCL1* and *SLC6A13*, the study employed the Just Another Spar Promoter Analysis Resource (JASPAR) database (http://jaspar.genereg.net/). JASPAR is a comprehensive repository that systematically compiles transcription factor binding profiles and matrices, offering valuable insights into the recognition motifs within gene promoter regions. Subsequently, the expression of *ASCL1* in TCGA-LIHC was detected using the Wilcox test method. Furthermore, R packages were used to examine the relationship between *ASCL1* and *SLC6A13* expression levels. It was determined that statistical significance was indicated by a *P* value of less than 0.05.

### Cell culture

Three HCC cell lines (Huh7, SNU-387, and MHCC-97H) and human liver normal cells (THL-2) were acquired from Shanghai Institutes for Biological Science, China, and were then cultured in Dulbecco’s Modified Eagle Medium (DMEM) mixed with 10% fetal bovine serum (FBS). Subsequently, the medium was supplemented with 100 µ/mL of penicillin and 100 µg/mL of streptomycin (Gibco) and kept at 37 ^∘^C with 5% CO_2_.

### Cell transfection and treatment assays

Gene-Pharma Co., Ltd. provided the vectors needed to overexpress *ASCL1* and *SLC6A13* (Shanghai, China). Based on established correlations in the HCC study, over-*ASCL1* and over-*SLC6A13* were transfected into SNU-387 and MHCC-97H cells [29]. Different doses of glycine (0, 0.05, 0.1, 0.25, 0.5, 1, 2, 4, 8 mM) and ethanol (0, 5, 15, 25, 40, 50 mM) were added to cells 24 h after transfection in order to better understand the cellular response to stress (the noticeable trend of a substantial increase prior to highlighting that the combination of 8 mM glycine and 50 mM ethanol yielded the highest results). Multiple tests were conducted to ensure reproducibility and reliability of the results.

### Quantitative real-time polymerase chain reaction (qRT-PCR) assay

After removing the total RNA from the cells using the Trizol reagent, PrimeScript^TM^ RT Master Mix (TAKARA, Dalian, China) was used to reverse transcribe the RNA into complementary DNA (cDNA). The FastStart Universal SYBR-Green Master Mix was used for qRT-PCR, and 2^−ΔΔCt^ was used to measure the relative mRNA levels of *ASCL1*, *SLC6A13*, IL-1β, interleukin-6 (IL-6), and TNF-α. Notably, gene expression data was normalized using glyceraldehyde-3-phosphate dehydrogenase (GAPDH) as an internal reference. The primers were designed in-house using Primer3 software based on the target gene sequences. The primer sequences used for *ASCL1*, *SLC6A13*, IL-1β, IL-6, TNF-α, and *GAPDH* are provided in Table 1.

**Table 1:** Primer sequences for qRT-PCR

**Target**	**Direction**	**Sequence**
*SLC6A13*	Forward	5′-GGGCATTGACAGCCAGTTCT-3′
*SLC6A13*	Reverse	5′-AAGTCTGGGGTACTCGTCCA-3′
*ASCL1*	Forward	5′-GAAGCAGGATGGCAGCAGAT-3′
*ASCL1*	Reverse	5′-TCGGGCTTAGGTTCAGACAC-3′
*IL-1β*	Forward	5′-ATGAAAGACGGCACACCCAC-3′
*IL-1β*	Reverse	5′-ATGAAAGACGGCACACCCAC-3′
*IL-6*	Forward	5′-GACAAAGCCAGAGTCCTTCAGA-3′
*IL-6*	Reverse	5′-TGTGACTCCAGCTTATCTCTTGG-3′
*TNF-α*	Forward	5′-AGGCACTCCCCCAAAAGAT-3′
*TNF-α*	Reverse	5′-TGAGGGTCTGGGCCATAGAA-3′
*GAPDH*	Forward	5′-TTGCCCTCAACGACCACTTT-3′
*GAPDH*	Reverse	5′-TGGTCCAGGGGTCTTACTCC-3′

qRT-PCR: Quantitative real-time polymerase chain reaction; ASCL1: Achaete-scute family bHLH transcription factor 1; IL-1β: Interleukin-1 beta; IL-6: Interleukin-6; TNF-α: Tumor necrosis factor-alpha; GAPDH: Glyceraldehyde-3-phosphate dehydrogenase.

### Western blotting (WB) assay

Protease and phosphatase inhibitors were included in the RIPA lysis solution (Thermo Fisher Scientific, USA), which was used to produce protein lysates from PC cells. The BCA Protein Assay Kit (Thermo Fisher Scientific, USA) was utilized to ascertain the protein content. Proteins in equal quantities were separated using 10% SDS-PAGE and then put onto PVDF membranes from Millipore, USA. Membranes were blocked with 5% skim milk and incubated with primary antibodies [*ASCL1*(Abcam, 1:3000), *SLC6A13*(Abcam, 1:200), IL-1β (Abcam, 1:1000), IL-6 (Abcam, 1:1000), TNF-α (Abcam, 1:1000), NLR family pyrin domain containing 1 (NALP1) (Abcam, 1:1000), absent in melanoma 2 (AIM2) (Abcam, 1:1000), NLRP3 (Abcam, 1:500), NLR family pyrin domain containing 4 (NLRC4) (Abcam, 1:100)] and appropriate secondary antibodies. As an internal reference, GAPDH (Abcam, 1:5000) was employed. Thermo Fisher Scientific, USA, provided an enhanced chemiluminescence (ECL) kit for visualizing protein bands, and Bio-Rad, USA, provided ChemiDoc imaging equipment for capturing the images.

### Dual luciferase assay

A dual-luciferase reporter assay was used to evaluate *ASCL1*-mediated transcriptional activity and its regulatory influence on *SLC6A13*. Firefly luciferase reporter constructs comprising wild-type (WT) or mutant (Mut) *SLC6A13* promoter sequences, as well as a Renilla luciferase vector as an internal control, were transiently transfected into 293T cells (from HanBio [Shanghai, China]). The constructs were cotransfected with the *ASCL1* expression vector to investigate whether *ASCL1* acts as an enhancer or repressor of *SLC6A13* transcription. After 48 h, the cells were separated, and a dual-luciferase reporter assay apparatus (Promega, Madison, WI, USA) was used to measure the amount of luciferase activity. To account for differences in transfection effectiveness, firefly luciferase values were adjusted to Renilla luciferase.

### Cell proliferation

The transfected cells were initially placed on a 96-well plate for the cell proliferation experiment. Each well plate was then filled with 10 µL of the Cell Counting Kit-8 (CCK-8) solution, and the cells were grown for a period of time (0, 24, 48, 72, 96 h) at 37 ^∘^C in a 5% CO_2_ atmosphere. After that, a microplate reader was used to measure the optical density (OD) values of the cells at 450 nm at various times.

### Cell migration and invasion

The transfected cells were inserted into the upper chamber of the Transwell after a certain volume of culture media had been supplied, and the bottom chamber was filled with RPMI-1640 medium containing 10% FBS. Matrigel (BD, USA) was used for invasion study but not for cell migration tests, where it was covered to the bottom. The extra cells on top were eliminated using a cotton swab, and the cells on the left were fixed in methanol for ten minutes before being stained with DAPI. Lastly, fluorescent microscopy was used to monitor cell invasion and migration in various areas.

### Enzyme-linked immunosorbent assay (ELISA)

Add appropriately diluted samples of cell culture supernatant to wells of an ELISA plate pre-coated with TNF-α, IL-1β, IL-6, gamma-glutamyltransferase 1 (GGT1), glutamate decarboxylase like 1 (GADL1), and histidine ammonia-lyase (HAL) antibodies. After the incubation and wash steps, the enzyme-linked secondary antibody is added, followed by the chromogenic substrate. The enzymatic reaction was halted at a designated time, and using a microplate reader, the absorbance was determined at the proper wavelength. Next, in order to determine the quantities of IL-1β, IL-6, TNF-α, GGT1, GADL1, and HAL, the absorbance values of the samples were compared to a standard curve made using the known concentrations of the pertinent standards.

### Statistical analysis

The language program R was used to carry out the statistical analysis. The KM method and the log-rank test were employed to examine survivability. Using Cox proportional hazards models, risk ratios (HR) and their 95% confidence intervals (CIs) were calculated. The data were analyzed using a one-way ANOVA for multiple comparisons and a two-tailed student’s *t*-test for group comparisons. The findings were expressed as mean ± SEM. *P* values were regarded as statistically significant if they were less than 0.05.

## Results

### Identification of DEGs and expression of *SLC6A13* in liver cancer

**Figure 1.**
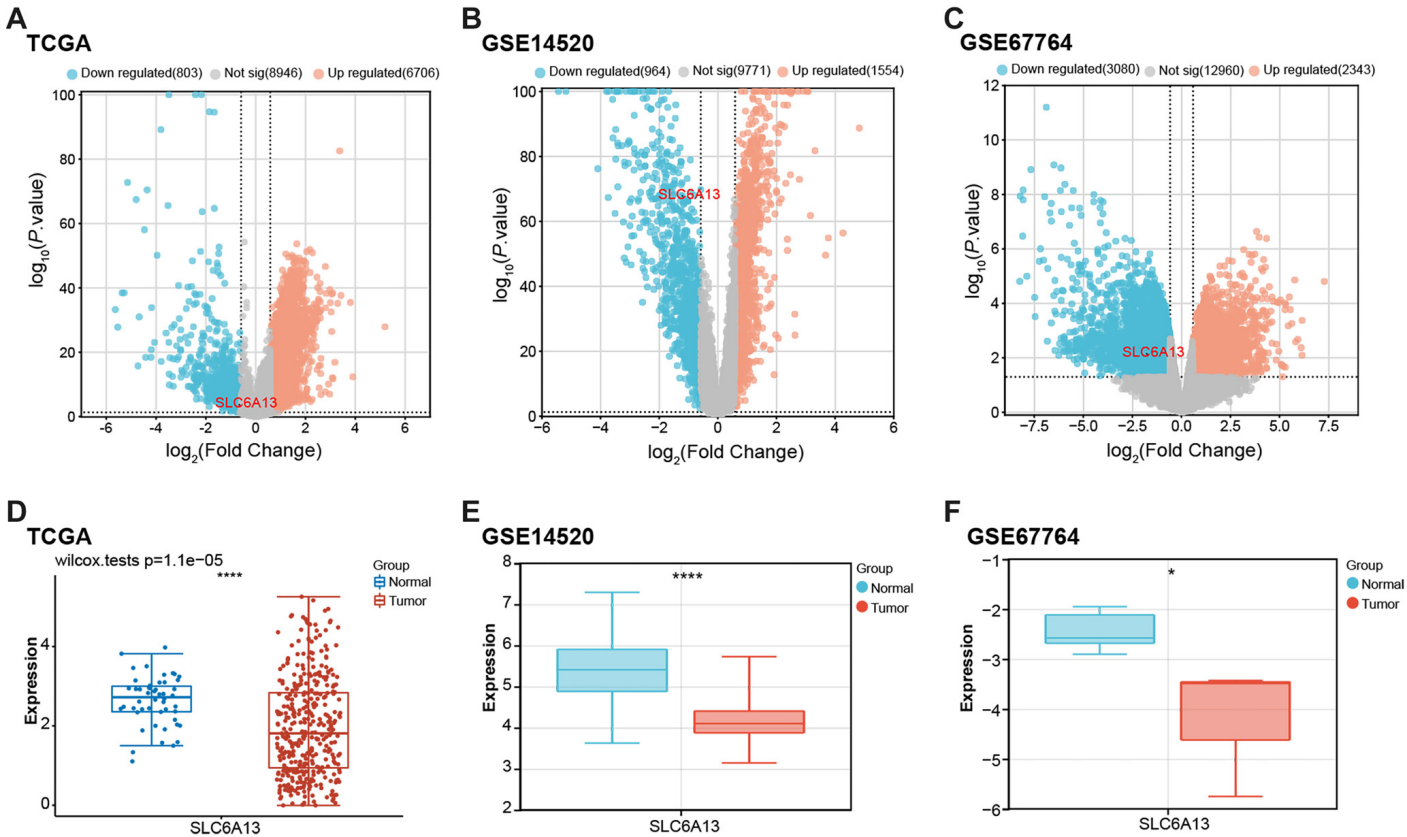
**Identification of DEGs and expression of *SLC6A13* in three datasets.** (A–C) Volcano plots of DEGs for the TCGA-LIHC dataset, GSE14520 dataset, and GSE67764 dataset. Orange represents upregulated genes, blue represents downregulated genes, and gray represents non-significant genes; (D–F) Box plots of *SLC6A13* expression analysis in TCGA-LIHC dataset, GSE14520 dataset, and GSE67764 dataset. Blue represents normal samples and red represents tumor samples. *P < 0.05, ****P < 0.0001. DEG: Differentially expressed genes; TCGA: The Cancer Genome Atlas; SLC6A13: Solute carrier family 6 member 13; TCGA-LIHC: The Cancer Genome Atlas-Liver Hepatocellular Carcinoma.

The TCGA-LIHC dataset produced 6706 upregulated and 803 downregulated genes based on predetermined selection criteria (Figure 1A). The GSE145290 dataset identified 1554 upregulated and 964 downregulated genes (Figure 1B), while the GSE67764 dataset revealed 2343 upregulated and 3080 downregulated genes (Figure 1C). Among them, *SLC6A13* was identified as a downregulated gene in all three datasets. Expression analysis further showed that the expression of *SLC6A13* was downregulated in tumor samples compared with normal samples in the TCGA-LIHC dataset (*P* ═ 1.1e^−05^), GSE145290 dataset, and GSE67764 dataset (Figure 1D–1F).

**Figure 2.**
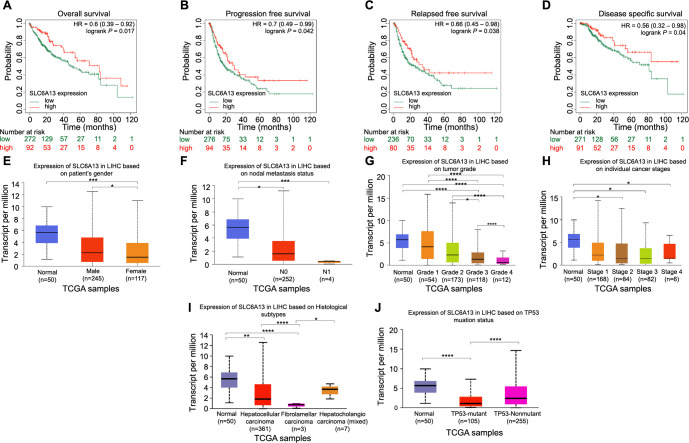
**Prognostic impact and clinicopathological correlation of *SLC6A13* expression in LIHC.** (A–D) KM survival curves of OS, PFS, RFS, and DSS of *SLC6A13*. Red represents high expression and green represents low expression; (E–J) Analysis of the expression characteristics of *SLC6A13* in patient’s gender, nodal metastasis status, tumor grade, individual cancer stages, Histological subtypes, and TP53 mutation status in UALCAN database. *P < 0.05, **P < 0.01, ***P < 0.001, ****P < 0.0001. KM: Kaplan–Meier; OS: Overall survival; PFS: Progression-free survival; RFS: Recurrence-free survival; DSS: Disease-specific survival; SLC6A13: Solute carrier family 6 member 13.

### Prognostic impact and clinicopathological relevance of *SLC6A13* expression in LIHC

By using KM survival curve analysis, the effect of *SLC6A13* expression on the prognosis of LIHC patients was investigated. Elevated *SLC6A13* expression has been linked to increased odds of OS, PFS, RFS, and DSS, as shown in Figure 2A–2D. Furthermore, UALCAN database analysis showed that *SLC6A13* expression was not affected by nodal metastasis status (Figure 2F) and individual cancer stages (Figure 2H). However, differential expression of *SLC6A13* was observed in different groups according to the patient’s gender (Figure 2E), tumor grade (Figure 2G), Histological subtypes (Figure 2I), and TP53 mutation status (Figure 2J).

### *ASCL1* transcriptional regulation of *SLC6A13*

**Figure 3.**
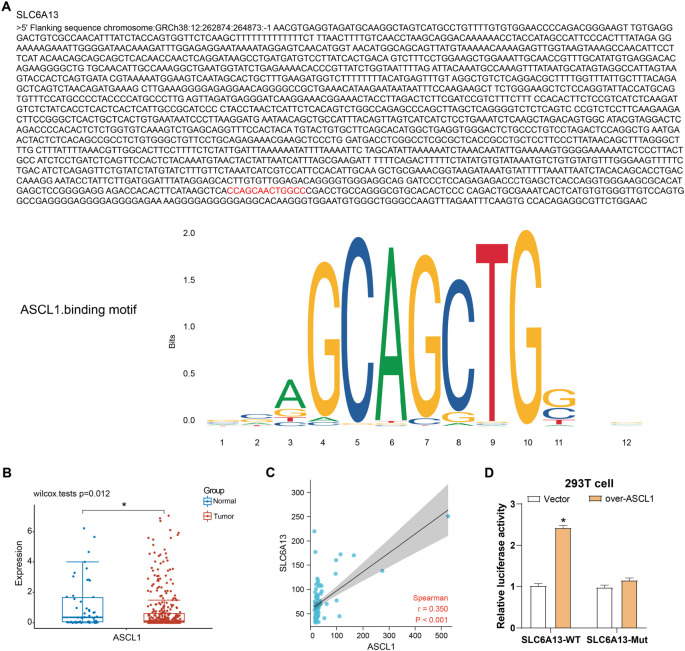
**Analysis of the interaction between *SLC6A13* and *ASCL1*.** (A) The red sequence is the binding site of *ASCL1* on the *SLC6A13* promoter; (B) Expression analysis of *ASCL1* in the TCGA-LIHC data set. Red represents tumor samples and blue represents normal samples; (C) Scatter plot, Spearman correlation analysis between *SLC6A13* and *ASCL1*; (D) Dual-luciferase assay measured luciferase activity in 293T cells transfected with WT or mutant (Mut) *SLC6A13* promoter constructs with or without co-transfection of the *ASCL1* overexpression vector. *P < 0.05. WT: Wild type; SLC6A13: Solute carrier family 6 member 13; ASCL1: Achaete-scute family bHLH transcription factor 1; TCGA-LIHC: The Cancer Genome Atlas-Liver Hepatocellular Carcinoma.

Analysis of the JASPAR database revealed *ASCL1* binding sites within the *SLC6A13* gene, suggesting that *ASCL1* has the potential to regulate *SLC6A13* expression through these binding sites. Subsequent verification of the TCGA database confirmed the expression of *ASCL1* in LIHC samples and normal tissues. The results showed that compared with normal liver tissue, *ASCL1* was significantly downregulated in LIHC samples (*P* ═ 0.012), indicating that *ASCL1* may be involved in tumorigenesis. The expression levels of *ASCL1* and *SLC6A13* exhibited a positive association (Spearman *r* ═ 0.350, *p* < 0.001), as demonstrated by the Spearman correlation analysis results, suggesting a regulatory interaction between the two in LIHC. Additionally, overexpression of *ASCL1* markedly increased the activity of the *SLC6A13*-WT promoter, as demonstrated by dual-luciferase analysis, while the activity of the mutant promoter was not enhanced, suggesting that *ASCL1* may regulate *SLC6A13* through specific binding sites (Figure 3D).

### *ASCL1* overexpression inhibits HCC cell proliferation, migration, and invasion

**Figure 4.**
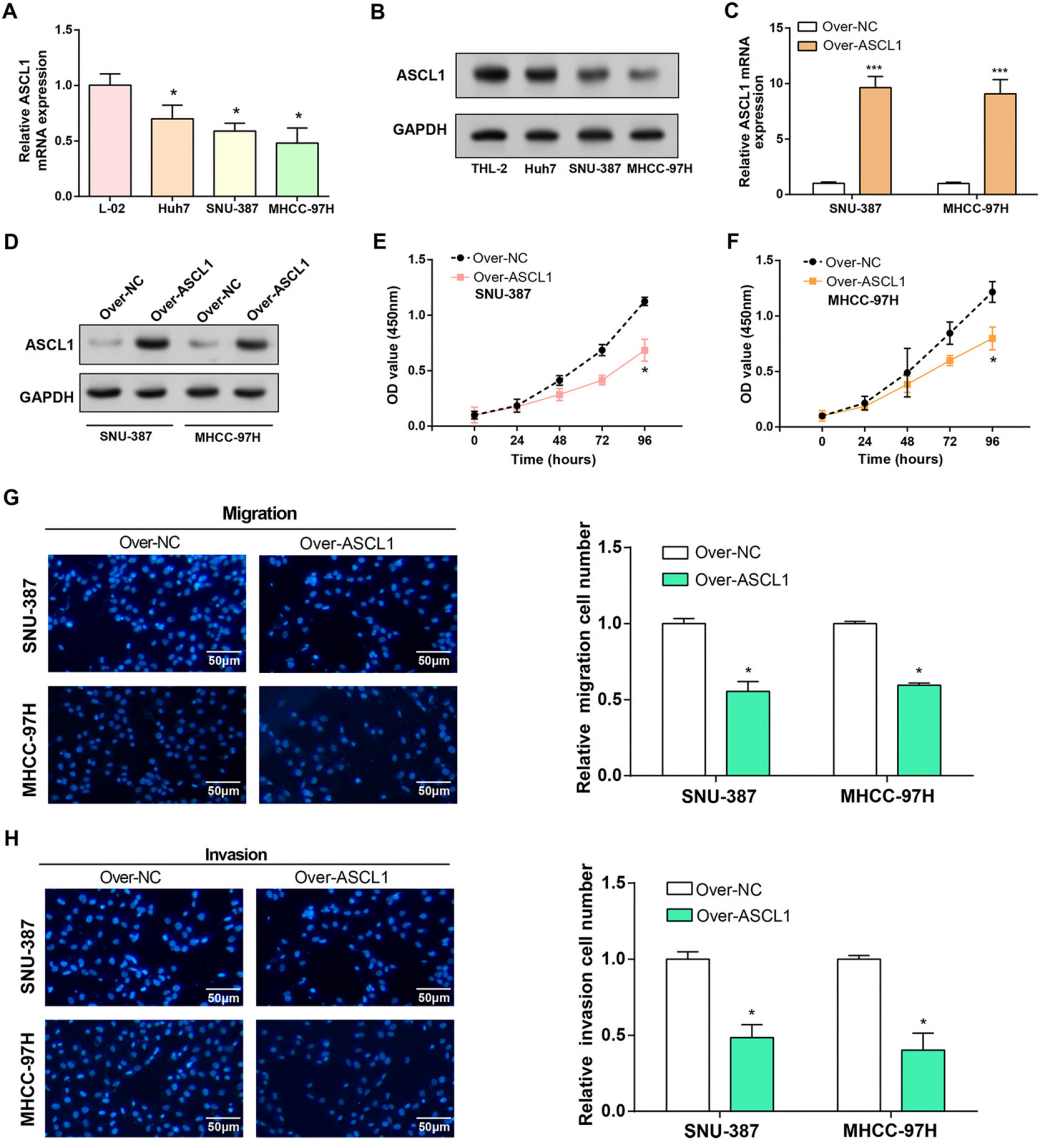
**Overexpression of *ASCL1* inhibits the proliferation, invasion, and migration of HCC cells.** (A and B) qRT-PCR and WB detected the expression of *ASCL1* in normal and HCC cell lines; (C and D) qRT-PCR and WB detected the overexpression efficiency of *ASCL1* in HCC cell lines (SNU-387 and MHCC-97H); (E and F) CCK-8 detected cell proliferation in HCC cell lines (SNU-387 and MHCC-97H) overexpressing *ASCL1*; (G and H) Transwell assay detected cell invasion and migration of overexpressed *ASCL1* in HCC cell lines (SNU-387 and MHCC-97H). The scale bar is 50 µm. *P < 0.05, ***P < 0.001. HCC: Hepatocellular carcinoma; WB: Western blotting; CCK-8: Cell-counting kit-8; qRT-PCR: Quantitative real-time polymerase chain reaction; ASCL1: Achaete-scute family bHLH transcription factor 1.

When *ASCL1* expression levels in HCC cell lines were examined, qRT-PCR and WB studies showed a significant downregulation, especially in SNU-387 and MHCC-97H cells (Figure 4A and 4B). Subsequently, efficient overexpression of *ASCL1* was confirmed at both mRNA and protein levels through qRT-PCR and WB assays, as illustrated in Figure 4C and 4D. The functional impact of *ASCL1* overexpression on HCC cell regulation was assessed by means of Transwell and CCK-8 investigations. SNU-387 and MHCC-97H cells overexpressing *ASCL1* had considerably less proliferation, invasion, and migration when compared to the control group (Figure 4E–4H).

### Overexpression of *SLC6A13* inhibits the proliferation, invasion, and migration of HCC cells

**Figure 5.**
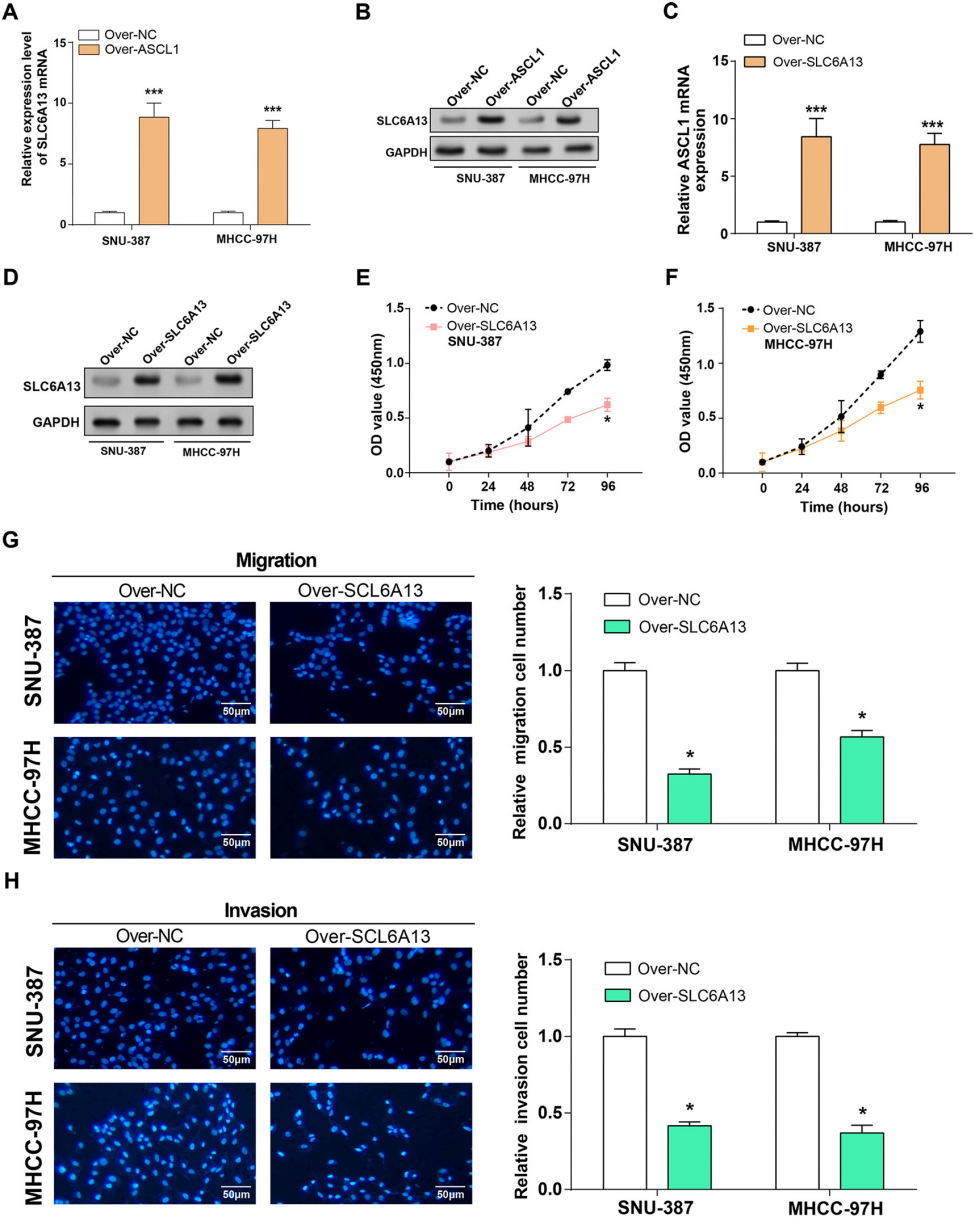
**Overexpression of *SLC6A13* inhibits the proliferation, invasion, and migration of HCC cells.** (A and B) qRT-PCR and WB detected the expression of *SLC6A13* in normal and HCC cell lines; (C and D) qRT-PCR and WB detected the overexpression efficiency of *SLC6A13* in HCC cell lines (SNU-387 and MHCC-97H); (E and F) CCK-8 detected cell proliferation in HCC cell lines (SNU-387 and MHCC-97H) overexpressing *SLC6A13*; (G and H) Transwell assay detected cell invasion and migration of overexpressed *SLC6A13* in HCC cell lines (SNU-387 and MHCC-97H). The scale bar is 50 µm. *P < 0.05, ***P < 0.001. HCC: Hepatocellular carcinoma; WB: Western blotting; CCK-8: Cell-counting kit-8; qRT-PCR: Quantitative real-time polymerase chain reaction; SLC6A13: Solute carrier family 6 member 13.

The mRNA and protein levels of *SLC6A13* were markedly elevated in SNU-387 and MHCC-97H cells overexpressing *ASCL1*, according to qRT-PCR detection, providing experimental evidence to support the regulatory relationship between *ASCL1* and *SLC6A13* (Figure 5A and 5B). Subsequently, efficient overexpression of *SLC6A13* was confirmed at both mRNA and protein levels through qRT-PCR and WB assays, as illustrated in Figure 5C and 5D. The regulatory impact of *SLC6A13* overexpression on HCC cell behavior was evaluated through CCK-8 and Transwell experiments. The overexpression of *SLC6A13* was observed to dramatically decrease the proliferation, invasion, and migration of SNU-387 and MHCC-97H cells in comparison to the control group (Figure 5E–5H).

### Overexpression of *SLC6A13* increases ethanol-induced inflammatory cytokine production

**Figure 6.**
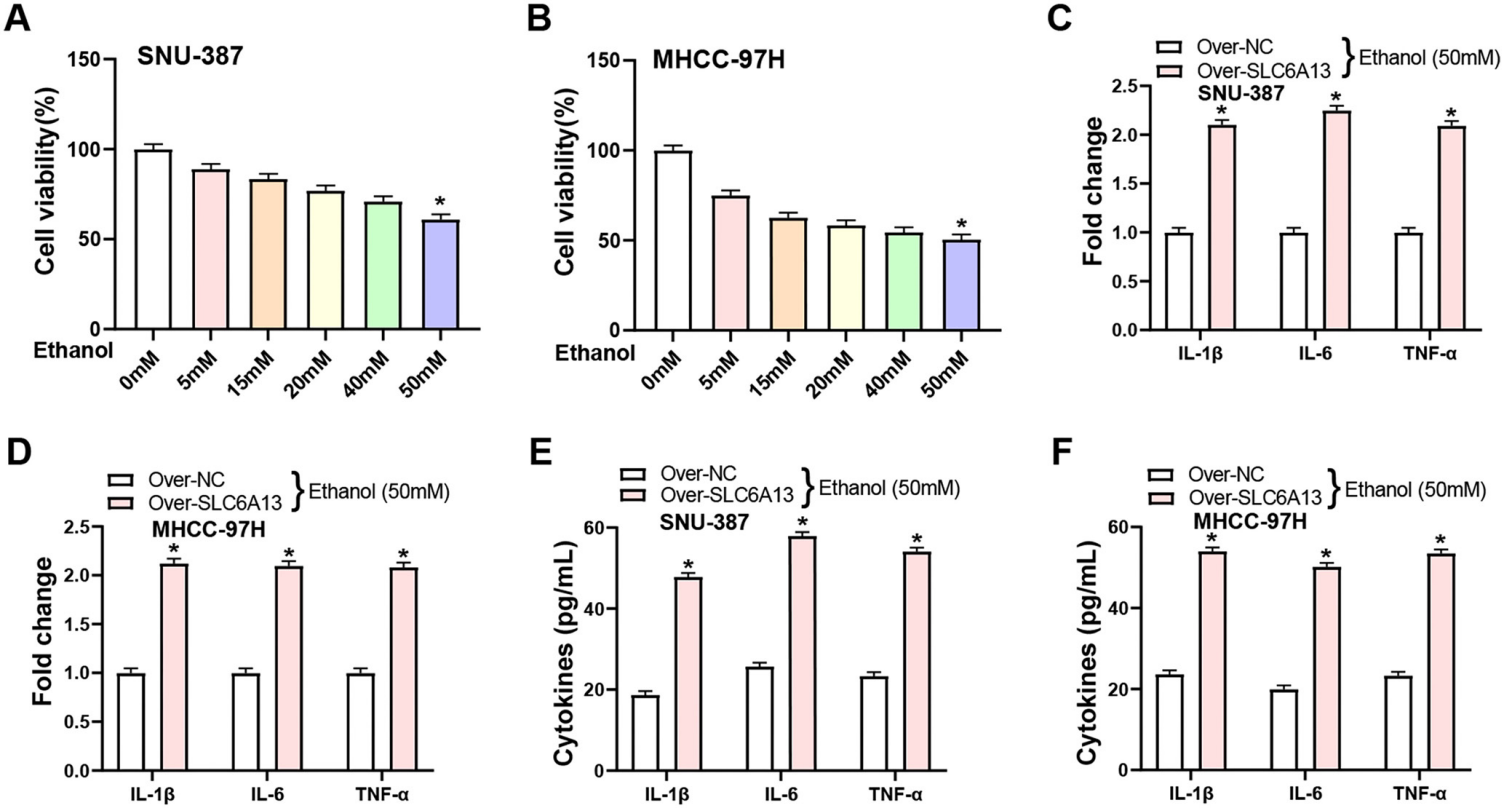
**Overexpression of *SLC6A13* increases ethanol-induced inflammatory cytokine production.** (A and B) CCK-8 detection on the viability of HCC cell lines (SNU-387 and MHCC-97H) after ethanol treatment at different concentrations (0, 5, 15, 25, 40, 50 mM) for 24 h; (C and D) qRT-PCR detected the effect of overexpression of *SCL6A13* on the expression of inflammatory factors (IL-1β, IL-6, and TNF-α) in HCC cell lines (SNU-387 and MHCC-97H) under 50 mM ethanol treatment; (E and F) ELISA experiment detected the effect of overexpression of *SCL6A13* on the secretion levels of inflammatory factors (IL-1β, IL-6, and TNF-α) in HCC cell lines (SNU-387 and MHCC-97H) under 50 mM ethanol treatment. *P < 0.05. CCK-8: Cell-counting kit-8; HCC: Hepatocellular carcinoma; ELISA: Enzyme-linked immunosorbent assay; SLC6A13: Solute carrier family 6 member 13; qRT-PCR: Quantitative real-time polymerase chain reaction; IL-1β: Interleukin-1 beta; IL-6: Interleukin-6; TNF-α: Tumor necrosis factor-alpha.

From the research of Nagappan et al. [30], we know that ethanol can induce an inflammatory response in HCC cell lines. To comprehend the potential toxicity of ethanol on HCC cell lines (SNU-387 and MHCC-97H), cells were treated with varying concentrations of ethanol (0, 5, 15, 20, 40, and 50 mM) for 24 h. CCK-8 assay results demonstrated that, among the tested ethanol doses, 50 mM induced significant cell death, as opposed to other concentrations (Figure 6A and 6B). qRT-PCR analysis showed that overexpression of *SLC6A13* significantly upregulated the expression levels of proinflammatory cytokines (IL-1β, IL-6, and TNF-α) in HCC cells following administration of 50 mM ethanol (Figure 6C and 6D). Protein levels were then confirmed by ELISA. Notably, *SLC6A13* overexpression induced by 50 mM ethanol treatment resulted in a significant increase in the secretion levels of IL-1β, IL-6, and TNF-α in HCC cell lines (Figure 6E and 6F).

### Glycine promotes inflammatory resistance to ethanol treatment by *SLC6A13* overexpression

**Figure 7.**
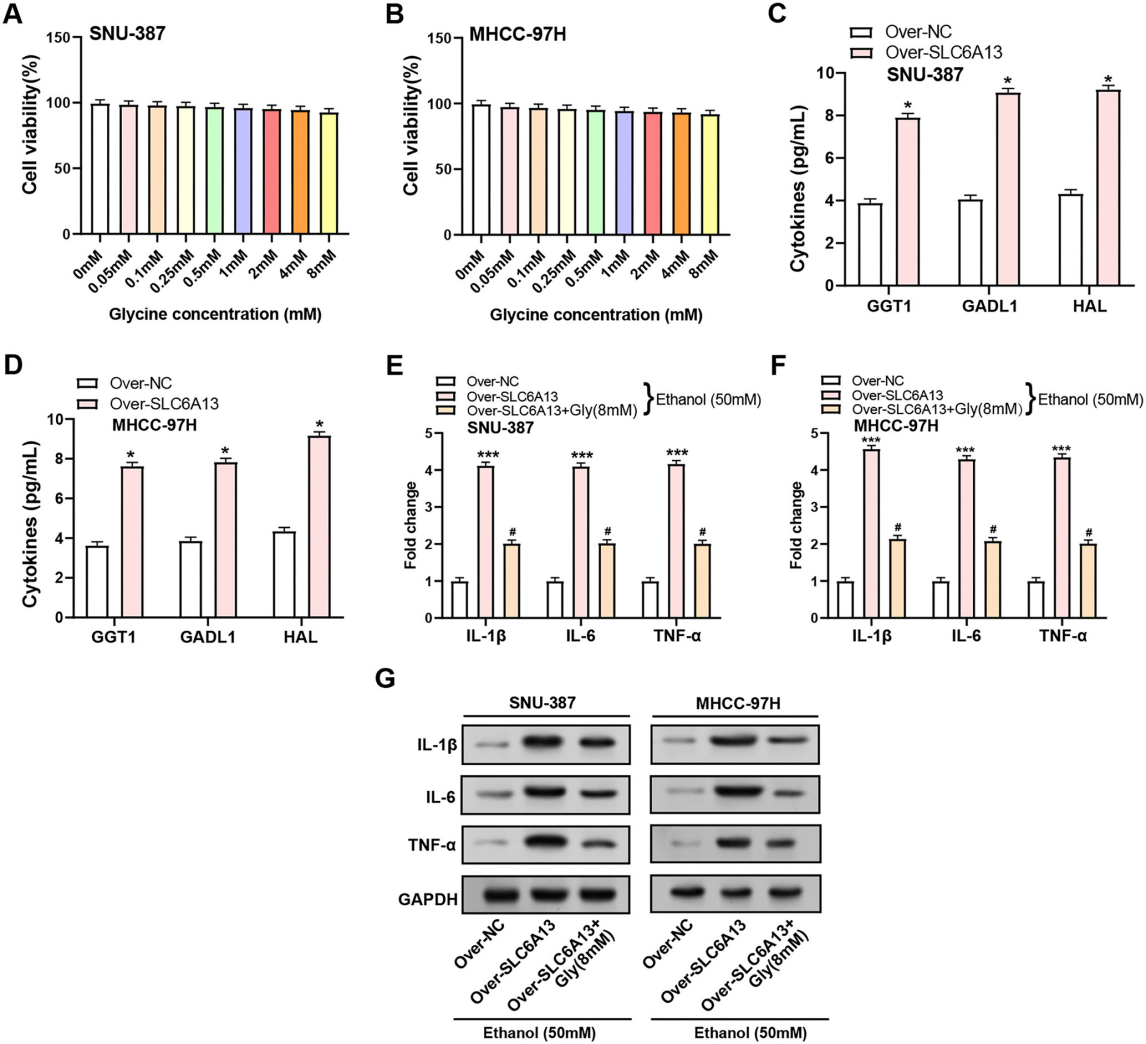
**Glycine promotes inflammatory resistance of *SLC6A13* overexpression to ethanol treatment.** (A and B) CCK-8 detected the effect of different concentrations of exogenous glycine on the cell viability of SNU-387 and MHCC-97H cells; (C and D) ELISA experiment detected the expression of amino acid metabolism-related genes after control and over-*SLC6A13* in SNU-387 and MHCC-97H cells; (E and F) qRT-PCR detection of inflammatory factors (IL-1β, IL-6, and TNF-α) expression in HCC cell lines (SNU-387 and MHCC-97H) after adding 50-mM ethanol for 24 h in three groups: Control and over-*SLC6A13*, over-*SLC6A13*+Gly (8 mM); (G) WB detection of inflammatory factors (IL-1β, IL-6, and TNF-α) expression in HCC cell lines (SNU-387 and MHCC-97H) after adding 50 mM ethanol for 24 h in three groups: Control and over-*SLC6A13*, over-*SLC6A13*+Gly (8 mM). *P < 0.05, ^#^*P* < 0.05, ***P < 0.001. CCK-8: Cell-counting kit-8; ELISA: Enzyme-linked immunosorbent assay; WB: Western blotting; HCC: Hepatocellular carcinoma; SLC6A13: Solute carrier family 6 member 13; qRT-PCR: Quantitative real-time polymerase chain reaction; IL-1β: Interleukin-1 beta; IL-6: Interleukin-6; TNF-α: Tumor necrosis factor-alpha.

Previous study had established that glycine, a nonessential amino acid, can attenuate inflammation induced by pathogenic bacteria and/or lipopolysaccharides (LPS) in various organs, such as the lungs, liver, and intestines [31]. In this study, the impact of exogenous glycine on the viability of HCC cell lines, SNU-387 and MHCC-97H, was assessed using CCK-8 assays. The findings demonstrated that the viability of HCC cells was not considerably impacted by varying glycine concentrations, suggesting that HCC cells were not exposed to any harmful effects (Figure 7A and 7B). Next, we looked at how the overexpression of *SLC6A13* affected the expression of GGT1, GADL1, and HAL, three enzymes involved in amino acid metabolism. The ELISA results indicated a notable increase in the expression levels of GGT1, GADL1, and HAL following the overexpression of SLC6A13, with each showing a more than twofold upregulation as shown in Figure 7C and 7D. Subsequent investigation utilizing qRT-PCR and WB showed that following treatment with 50 mM ethanol for 24 h, *SLC6A13* overexpression led to a substantial increase in the production of inflammatory markers IL-1β, IL-6, and TNF-α. Interestingly, the addition of glycine (8 mM) partially reversed this upregulation, highlighting a potential regulatory role of glycine in the inflammatory response triggered by *SLC6A13* overexpression (Figure 7E–7G).

### Regulation of inflammasome activation by *SLC6A13* and *ASCL1* in HCC cell lines treated with ethanol and glycine

**Figure 8.**
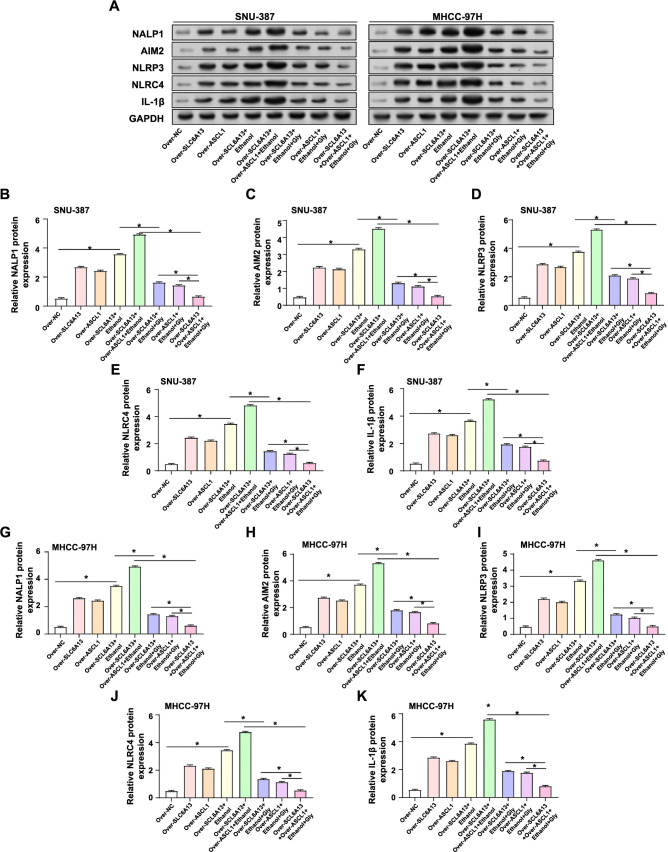
**Expression analysis of inflammasome components in SNU-387 and MHCC-97H cells**. (A–K) WB assays were performed to detect the expression levels of inflammasome-related proteins, including NALP1, AIM2, NLRP3, NLRC4, and IL-1β, in different groups (Over-NC, Over-*SLC6A13*, Over-*ASCL1*, Over-*SCL6A13*+Ethanol, Over-*SCL6A13*+Over-ASCL1+Ethanol, Over-*SLC6A13*+Ethanol+Gly, Over-*ASCL1*+Ethanol+Gly, Over-*SCL6A13*+Over-*ASCL1*+Ethanol+Gly) of SNU-387 and MHCC-97H cell lines. *P < 0.05. WB: Western blotting; NALP1: NLR family pyrin domain containing 1; AIM2: Absent in melanoma 2; NLRP3: NLR family pyrin domain containing 3; NLRC4: NLR family pyrin domain containing 4; IL-1β: Interleukin-1 beta.

Study on inflammasome activation had shown that overexpression of *SLC6A13* in liver cancer cell lines regulates the expression of key components of the inflammasome [32]. WB analysis revealed differential expression patterns of inflammasome components after various treatments in HCC cell lines. As shown in Figure 8A–8K, overexpression of *SLC6A13* alone or overexpression of *ASCL1* alone resulted in upregulation of protein levels of inflammasomes (NALP1, AIM2, NLRP3, and NLRC4) as well as the inflammatory factor (IL-1β). The expression of these proteins was further enhanced when combined with ethanol (50 mM) treatment. Furthermore, co-overexpression of *SLC6A13* and *ASCL1* followed by ethanol treatment resulted in the highest observed expression levels of these proteins, suggesting a synergistic effect on the upregulation of inflammasome components. Notably, in cells overexpressing *SLC6A13* or *ASCL1* alone, ethanol (50 mM) treatment and addition of glycine (8 mM) significantly downregulated the expression of the detected proteins. This inhibitory effect was most pronounced in cells co-overexpressing *SLC6A13* and *ASCL1* and treated with both ethanol (50 mM) and glycine (50 mM). Taken together, our results highlighted the reciprocal regulatory role between *SLC6A13* and *ASCL1* on inflammatory factors in the presence of ethanol and glycine.

## Discussion

HCC is the main pathological type of primary liver cancer and is the second most lethal cancer worldwide, with the fourth highest incidence and third highest mortality. The prognosis for advanced HCC is very unfavourable, with only about 30% of resected patients and 60% of transplanted patients surviving for five years. Chronic hepatitis B or C virus infection (HBV/HCV), type 2 diabetes mellitus, and smoking are the main risk factors for HCC [33]. Despite some advances in diagnosis and treatment in recent years, such as transcatheter arterial chemoembolisation and the introduction of the multikinase inhibitor sorafenib, the survival rate of HCC remains very low due to the challenge of high recurrence and metastasis rates [34]. Both the incidence and mortality of HCC have been trending upward in recent years. Although the prognosis is not good, liver resection and liver transplantation are still the major therapies for HCC [35]. The field of bioinformatics is still developing nowadays, which is helping to enhance the creation of biomarkers. These biomarkers and genetic models provide new directions for assessing patient prognosis and making treatment decisions about tumor-specific changes. The study had found genes related to the prognosis of HCC, including DEAD-box helicase 24 (*DDX24*), cleavage and polyadenylation specific factor 2 (*CPSF2*), tetratricopeptide repeat domain 2*6* (*TTC26*), TATA-box binding protein associated factor 3 (*TAF3*), alpha fetoprotein (*AFP*), etc. These genes can affect the biological behavior of HCC through different mechanisms, including cell proliferation, apoptosis, invasion, and metastasis. Therefore, understanding the role of prognostic genes is crucial to improve the treatment of HCC. The results of our bioinformatics in our study on TCGA-LIHC, GSE14520, and GSE67764 datasets showed that *SLC6A13* expression was continuously downregulated in liver cancer samples. Further prognostic analysis found that increased *SLC6A13* expression was associated with better prognosis. Furthermore, clinical feature analysis results confirmed that the expression of *SLC6A13* is affected by different clinical parameters of LIHC, especially in terms of tumor grade, gender differences, histological subtypes, and TP53 mutation status. These findings highlight the potential of *SLC6A13* as a biomarker for HCC and its potential prognostic value in HCC patients.

The basic helix-loop-helix (bHLH) family of transcription factors, which includes the *ASCL1* gene, has been the subject of much research due to its diverse function in cancer biology and developmental processes [36]. The several roles of *ASCL1* in the realm of cancer have been made clear by a study by Zhu et al. Specifically, *ASCL1* exhibits significantly higher expression in pure small-cell lung carcinoma (P-SCLC) compared to combined small-cell lung carcinoma (C-SCLC), suggesting its potential correlation with histological subtypes of C-SCLC [37]. Furthermore, in the study by Zhu et al. [38], *ASCL1* is identified as a key component of a prognostic signature associated with breast cancer survival, emphasizing its role in influencing outcomes through microenvironment-associated mechanisms. Building upon the understanding of *ASCL1* in cancer biology, the study by Zhou et al. [39] explores its critical role in NE differentiation and proliferation within small cell lung cancer (SCLC) tissues from patients with non-small cell lung adenocarcinoma (LUAD) resistant to EGFR tyrosine kinase inhibitors (TKIs). It has been demonstrated that in androgen-dependent prostate cancer cells, upregulation of MUC1-C inhibits androgen receptor (AR) signaling and drives the expression of the neurotranscription factor BRN2. Activation of MUC1-C induces molecular markers associated with the progression of NE prostate cancer (NEPC) through the MYC→BRN2 pathway, including NE differentiation markers *ASCL1* [40]. Two molecular subtypes, the PSCCE-A and PSCCE-N subtypes of primary small cell carcinoma of the esophagus (PSCCE), have been defined by different gene expression patterns regulated by ASCL1. These subtypes are highly similar to those of SCLC at the molecular level [41]. Transcriptomic analyses confirmed previously described subtypes based on the expression of genes such as ASCL1 and revealed an additional dimension of clinical subtypes that encompassed mixed NE and non-NE phenotypes. Characteristics of these subtypes include resistance to chemotherapy and poor prognosis [42]. Building upon these insights, our raw letter analysis and experimental results delved into the significance of *ASCL1* in HCC. The JASPAR database confirmed the binding of *ASCL1* to *SLC6A13*, and *ASCL1* was significantly overexpressed in normal samples in the TCGA-LIHC dataset and positively correlated with *SLC6A13*. Dual-luciferase assay verified the transcriptional regulatory effect of *ASCL1* on *SLC6A13*. Based on in vitro investigations, *ASCL1* functions as a tumor suppressor in HCC cells, preventing the growth, migration, and invasion of cancer cells. Subsequent investigation revealed that *SLC6A13* expression was markedly increased by *ASCL1* overexpression. Furthermore, *SLC6A13* also strongly suppressed the cancerous activity of HCC cells, suggesting that *ASCL1* and *SLC6A13* may be useful therapeutic targets for HCC.

The intricate interactions between ethanol, inflammatory responses, inflammatory cytokines (IL-1β, IL-6, TNF-α), and enzymes related to amino acid metabolism (GGT1, GADL1, HAL) emphasize the complex physiological consequences of ethanol consumption. Alcoholic beverages include ethanol, a psychoactive compound that depresses the central nervous system and affects the body as a whole through hepatic metabolism [43, 44]. Inflammatory responses are integral to ethanol-induced effects, with cytokines modulating inflammation and coordinating immune responses [45]. Glycine is a non-essential amino acid with anti-inflammatory properties that can reduce inflammation caused by disease-causing agents in various organs. A specific study examined by Nagappan et al. demonstrated that ethanol induces inflammatory responses in liver cancer cell lines. Furthermore, glycine decarboxylase (GLDC) plays a critical role in the development and metastasis of HCC, according to research by Zhuang et al. [46]. GLDC downregulation is mediated by miRNA-30d-5p, affects autophagy, and promotes HCC progression. Furthermore, GLDC knockdown enhanced reactive oxygen species (ROS), reduced cofilin ubiquitination, and promoted HCC cell migration and invasion. Enzymes related to amino acid metabolism play multiple roles in cancer biology, affecting tumor occurrence, growth, and metastasis. GGT1 is an enzyme widely present on cell membranes, involved in the metabolism of glutathione (GSH), and related to the occurrence, development, and drug resistance of tumors [47]. Few investigations have been done on GADL1, which may be involved in controlling how glutamate and related amino acids are metabolized. HAL is involved in histidine metabolism and can convert histidine into uric acid, which is related to biological processes, such as cell proliferation and immune response [48]. Understanding the intricate network of metabolic pathways within cells is crucial for unraveling the complex mechanisms underlying various biological processes. Our experimental results showed that in HCC cells, overexpression of *SLC6A13* significantly upregulated the expression of GGT1, GADL1, and HAL. This suggested that *SLC6A13* may play an important role in regulating key enzymes related to amino acid metabolism, thereby affecting the metabolic status of HCC cells and the tumor microenvironment. Moreover, 50 mM ethanol markedly increased cell death and cytokine expression, and overexpression of *SLC6A13* in HCC cell lines improved ethanol-induced production of inflammatory cytokines, such as IL-1β, IL-6, and TNF-α. In contrast, glycine treatment attenuated this inflammatory response, suggesting a modulatory role in ethanol-induced inflammation.

Additionally, we analyzed the role of inflammasome in HCC. NALP1 is part of the NLRP1 inflammasome, which can induce the maturation and secretion of IL-1β, activate inflammatory responses, and promote tumor growth and spread [49]. AIM2 is an intracellular DNA sensor that triggers inflammatory reactions [50]. Ectopic expression of AIM2 in HCC cells has been demonstrated to drastically impede migration and increase apoptosis. The NLRP3 inflammasome is involved in numerous inflammatory disorders. NLRP3 activation in HCC may be related with hepatocyte destruction and liver fibrosis, accelerating the course of the disease [51]. The activation of the NLRC4 inflammasome can also boost the production of inflammatory substances in HCC and influence tumor cell malignant development [52]. IL-1β is also a major effector molecule in the inflammasome pathway, regulating and activating inflammatory responses as well as encouraging tumor cell proliferation [53]. Uncover the intricate interplay of these factors in the inflammatory response and tumor cell proliferation. Our results indicated that co-overexpression of *SLC6A13* and *ASCL1* further amplified levels of inflammasome components, including NALP1, AIM2, NLRP3, NLRC4, and IL-1β in response to ethanol. This synergy was inhibited by glycine, highlighting the complex interplay of *SLC6A13*, *ASCL1,* and glycine in regulating the inflammatory response in HCC.

## Conclusion

To sum up, our study elucidated a complex network involving *SLC6A13*, *ASCL1,* and glycine during HCC progression. It was discovered that *SLC6A13* was downregulated in HCC samples and that individuals with LIHC had a better prognosis when its expression was elevated. The research also showed that *ASCL1*, which had a positive correlation with *SLC6A13*, controls the production of this protein and suppresses the growth, migration, and invasion of HCC cells, hence functioning as a tumor suppressor. Similarly, overexpression of *SLC6A13* inhibited the malignant behavior of HCC cells, thus providing a potential avenue for therapy. Furthermore, overexpression of *SLC6A13* upregulated key amino acid metabolizing enzymes (GGT1, GADL1, HAL) and enhanced ethanol-induced inflammatory cytokine production, which was modulated by glycine, suggesting a complex interaction in the inflammatory response in HCC. The synergistic effect of *SLC6A13* and *ASCL1* in regulating inflammasomes (NALP1, AIM2, NLRP3, NLRC4) and inflammatory factors (IL-1β) highlighted their important role in the pathogenesis and progression of HCC. By shedding light on the molecular origins of HCC, this study offers potential targets for future therapies aimed at *SLC6A13* and *ASCL1*.

## Data Availability

The datasets used and/or analyzed during the current study are available from the corresponding author on reasonable request.
